# mTORC1 Signaling Promotes Osteoblast Differentiation from Preosteoblasts

**DOI:** 10.1371/journal.pone.0130627

**Published:** 2015-06-19

**Authors:** Jianquan Chen, Fanxin Long

**Affiliations:** 1 Department of Orthopedics, The First Affiliated Hospital of Soochow University, Suzhou, Jiangsu, China; 2 Orthopedic Institute, Soochow University, Suzhou, Jiangsu, China; 3 Department of Orthopedic Surgery, Washington University, St. Louis, Missouri, United States of America; 4 Department of Medicine, Washington University, St. Louis, Missouri, United States of America; 5 Department of Developmental Biology, Washington University, St. Louis, Missouri, United States of America; Georgia Regents University, UNITED STATES

## Abstract

Preosteoblasts are precursor cells that are committed to the osteoblast lineage. Differentiation of these cells to mature osteoblasts is regulated by the extracellular factors and environmental cues. Recent studies have implicated mTOR signaling in the regulation of osteoblast differentiation. However, mTOR exists in two distinct protein complexes (mTORC1 and mTORC2), and the specific role of mTORC1 in regulating the progression of preosteoblasts to mature osteoblastis still unclear. In this study, we first deleted *Raptor*, a unique and essential component of mTORC1, in primary calvarial cells. Deletion of *Raptor* resulted in loss of mTORC1 but an increase in mTORC2 signaling without overtly affecting autophagy. Under the osteogenic culture condition, *Raptor*-deficient cells exhibited a decrease in matrix synthesis and mineralization. qPCR analyses revealed that deletion of Raptor reduced the expression of late-stage markers for osteoblast differentiation (*Bglap*, *Ibsp*, and *Col1a*), while slightly increasing early osteoblast markers (*Runx2*, *Sp7*, and *Alpl*). Consistent with the findings *in vitro*, genetic ablation of *Raptor* in osterix-expressing cells led to osteopenia in mice. Together, our findings have identified a specific role for mTORC1 in the transition from preosteoblasts to mature osteoblasts.

## Introduction

Preosteoblasts are a heterogeneous population of progenitor cells that are committed to the osteoblast lineage [1, 2]. Preosteoblasts are derived from multi potent mesenchymal progenitor cells (MPs), and can further differentiate along the osteoblast lineage into mature osteoblasts (the major bone-forming cells) in response to osteogenic signals. The identities of preosteoblasts are still not fully understood, but they initially express the transcription factor *Runx2*, and then both *Runx2* and Sp7 (*Osterix)* [2]. The process of osteoblast differentiation, including transitioning of preosteoblasts into mature osteoblasts, is tightly regulated by extracellular factors and environmental cues either positively or negatively [2].

The mechanistic target of rapamycin (mTOR) pathway is an evolutionally conserved nutrient-sensing pathway that controls many major cellular processes [3]. mTOR is a serine/threonine kinase, which exists in two different complexes (mTORC1 and mTORC2). These two complexes can be distinguished by their specific and essential components, such as Raptor for mTORC1 and Rictor for mTORC2. Activated by upstream signals, mTORC1 and mTORC2 control different downstream effectors and cellular processes [4]. Genetic deletion of raptor or rictor in the early embryonic mesenchyme in the mouse has revealed important roles formTORC1 and mTORC2 in skeletal development. mTORC1 is crucial for the regulation of chondrocyte size, hypertrophy and matrix production [5], whereasmTORC2 enhances bone formation [6]. These studies, however, did not address the role of mTOR specifically in preosteoblasts.

Recently, the mTOR pathway has been also implicated in controlling MPs fate and osteoblast differentiation. Studies using mTOR inhibitor rapamycin or mTOR gene ablation showed that mTOR signaling could exert both stimulatory [7, 8, 9, 10, 11] and inhibitory [12, 13, 14, 15] effects on osteoblast differentiation. These conflicting results may reflect stage-specific roles of mTOR within the osteoblast lineage. Moreover, since prolonged treatment with rapamycinor gene deletion of mTOR inhibits both mTORC1 and mTORC2pathways [3], the specific role of mTORC1 in osteoblast differentiation remains unclear.

In the current study, by using the Cre/LoxP technology to delete *Raptor* in primary cultures of calvarial preosteoblasts, orin osterix-expressing cells in mice, we demonstrate a critical role formTORC1 in the transition of preosteoblasts to mature osteoblasts.

## Materials and Methods

### Mouse strains

*Osx-Cre* and *Raptor* ^*f/f*^ mouse lines were previously reported [16, 17] and were obtained from Jackson Laboratory (Bar Harbor, ME). This study was performed in strict accordance with the recommendations in the Guide for the Care and Use of Laboratory Animals of the National Institutes of Health. The protocol in this study was approved by Animal Studies Committee at Washington University. All efforts were made to minimize animal suffering.

### X-ray radiography and μCT

Six week-old mice were euthanized by trained personnel using carbon dioxide, and then limbs were collected for X-ray and μCTanalyses. X-ray radiography was conducted on hind limbs with a Faxitron X-ray system at 25 kv for 20 seconds. μCT analyses were performed with Scanco μCT 40 (Scanco Medical AG) according to ASBMR guidelines [18]. 100 μCT slices (1.6 mm total) immediately below the growth plate of the tibias were used for 3D reconstruction and quantification of trabecular bone parameters.

### Primary calvarial cell isolation and adenovirus infection

To isolate calvarial cells, newborn *Raptor* ^*f/f*^ pups were euthanized by decapitation with sharp scissors. Subsequently, the frontal and parietal bones (FPB) were dissected, rinsed with PBS, and then subjected to five serial digestions with 1.8 mg/ml collagenase (Sigma). The first digestion was discarded and digestions two to five were pooled and then filtered through a 70 μm cell strainer. Collected cells were seeded in 12-well plate sat 1.5×10^5^ cells/well. After overnight culture, cells were infected with adenovirus expressing either green fluorescence protein (Ad-GFP) or Cre(Ad-CRE) at a multiplicity of infection of 50. At 72 h after adenoviral infection, cells were either harvested for protein analysis or induced for osteoblast differentiation by osteogenic medium (αMEM media with 10% FBS, 10mM β-glycerol phosphate and 50μg/ml ascorbic acid). Osteogenic medium was changed every other day.

### Mouse bone marrow stromal cell (BMSC) cultures and osteogenic differentiation

Six week-old RapCKO or control mice were euthanized by carbon dioxide method prior to isolation of BMSCs. Isolation, culture, and osteogenic differentiation of BMSCs were performed as previously described [6].

### Alkaline phosphatase (AP) staining and Von Kossa Staining

For AP staining, cells were fixed with 3.7% formaldehyde, rinsed with PBS, and then incubated with a reaction mixture (0.1 mg/ml naphthol AS-MX phosphate,0.6 mg/ml Fast blue BB Salt,0.5% N, N-demethylformamide, 2 mM MgCl2, and 0.1 M Tris/HCl) at room temperature for 20 minutes. For Von Kossa staining, cells were fixed in cold methanol, rinsed with distilled water, and then incubated with 5% silver nitrate solution under bright light for 30 minutes. Excess stain was washed off with double distilled water.

### Quantitative real-time PCR (qPCR) and Western blot

Total RNA was extracted from cells using the QiagenRN easy kit following the manufacturer’s instructions. One microgram of isolated RNA was reverse transcribed to cDNA using the iScriptcDNA synthesis kit (Bio-Rad) according to the manufacturer’s protocols. qPCR was conducted with SYBR-Green Supermix (Bio-Rad). Relative gene expression was determined by first normalization to β-actin, and then normalization to control samples.

Western blot analyses were performed as previously described [5]. Antibodies for S6,pS6 (S240/244), Akt, pAKT (S473), Raptor, Rictor, LC3B, and β-actin were all purchased from Cell Signaling Technology. All antibodies were used at 1:1000.

### Enzyme-Linked Immunosor bent Assay (ELISA) for P1NP

P1NP levels in the culture medium of calvarial cells were determined by Enzyme-Linked Immunosor bent Assay (ELISA) using the Rat/Mouse P1NP EIA kit (Immunodiagnostic Systems, Ltd.) according to the manufacturer’s recommendation.

### Statistics

All quantitative data are presented as mean ± STDEV with a minimum of three independent samples. Statistical significance is determined by two-tailed Student’s t-test. P-value less than 0.05 is considered statistically significant.

## Results

### Deletion of *Raptor* in preosteoblasts disruptedmTORC1 but activated mTORC2 signaling

**Fig 1:**
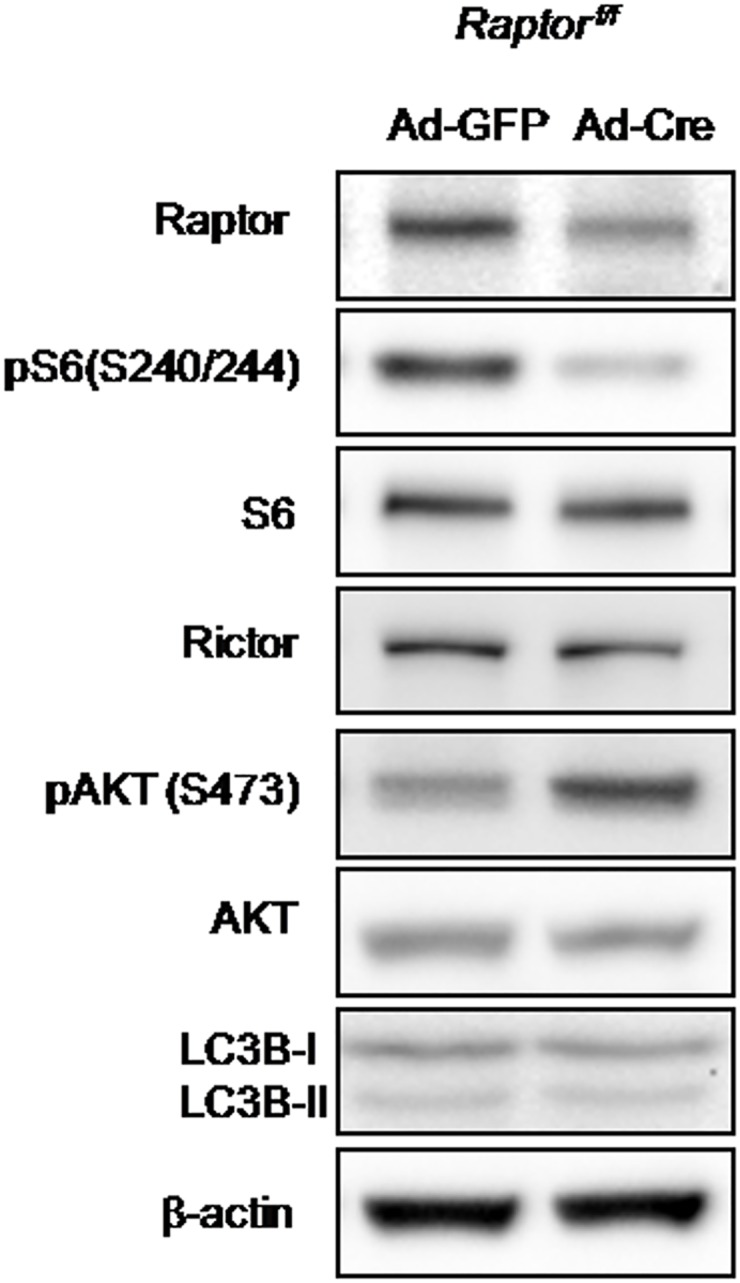
Deletion of *Raptor* in preosteoblasts disrupted mTORC1 but activated mTORC2 signaling. Western blot analyses of primary calvarial cells isolated from *Raptor* ^*f/f*^neonates and infected with adenoviruses expressing GFP (Ad-GFP) or Cre (Ad-Cre) for 3 days.

To determine the specific role of mTORC1 in osteoblast differentiation, we isolated primary osteoblast precursors from the calvarium of neonatal *Raptor* ^*f/f*^ mouse, and infected these cells with adenoviruses expressing GFP (Ad-GFP) or Crerecombinase (Ad-Cre). Western blot analysis showed that Raptor protein was efficiently deleted 3 days after adenovirus infections (Fig 1). As expected, Rictor protein was intact in Ad-Cre infected cells (Fig 1). We then examined the effects of *Raptor* deletion on mTORC1 and mTORC2 activity. Western blot showed that phosphorylation of S6 at sites of S240/244 (a readout of mTORC1 activity) was dramatically reduced, while phosphorylation of Akt at S473 (a specific target of the mTORC2 complex) was significantly increased (Fig 1). These data indicated that deletion of *Raptor* led to impaired mTORC1 activity and enhanced mTORC2 activity. In addition, mTORC1 signaling is known to suppress autophagy in many cell types [3]. Because autophagy has been shown to regulate osteoblast differentiation and activity [19, 20], we asked whether deletion of *Raptor* in preosteoblast cells activated autophagy. To this end, we analyzed LC3B isoforms (LC3B-I and LC3B-II) by Western blot, since the conversion of LC3-I to the lower migrating form, LC3-II, is commonly used as an indicator of autophagy [21]. There was no difference in either LC3B-I or LC3B-II between WT and *Raptor*-deleted cells, indicating that disruption of Raptor/mTORC1 does not activate autophagy in the preosteoblasts.

### Deletion of *Raptor* in preosteoblasts reduced matrix synthesis and mineralization

**Fig 2:**
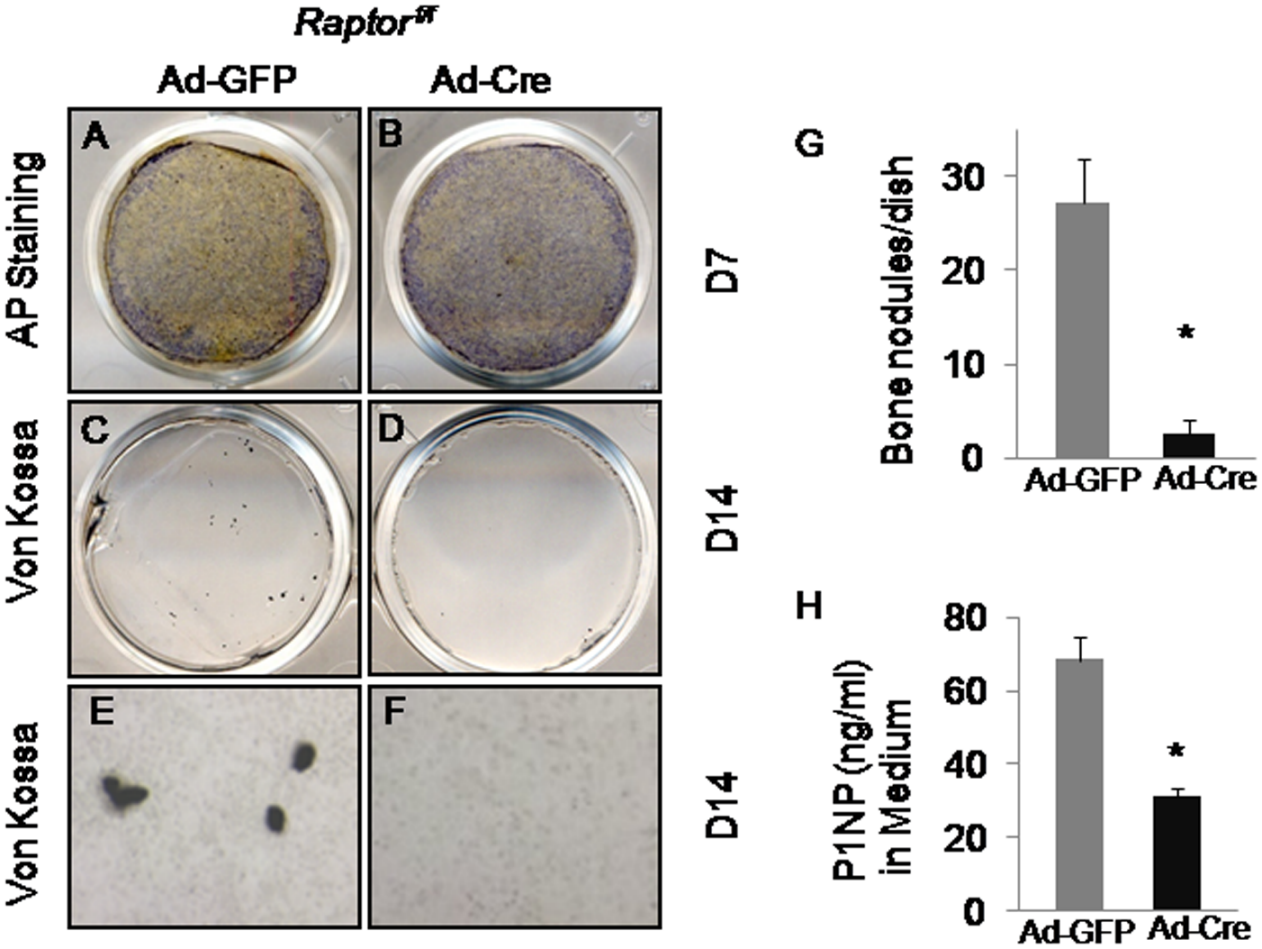
Deletion of *Raptor* in preosteoblasts reduced matrix synthesis and mineralization. (A-B) Alkaline phosphatase (AP) staining of wildtype (Ad-GFP)(A) versus *Raptor*-deficient (Ad-Cre)(B) calvarial cells after 7 days (D7) of osteogenic differentiation. (C-F)Von Kossastaining of wildtype (Ad-GFP)(C, E) versus *Raptor*-deficient (Ad-Cre)(D, F) calvarial cells after 14 days (D14) of osteogenic differentiation. Panels C-D showed lower magnification images; Panels E-F showed higher magnification images. (G) Quantification of bone nodules detected by Von Kossa staining. n = 3; *:P<0.05. (H) ELISA analysis of P1NP levels in wildtype (Ad-GFP) versus *Raptor*-deficient (Ad-Cre) calvarial cells after 14 days (D14) of osteogenic differentiation. n = 3; *:P<0.05.

We next assessed the capability of *Raptor*-deficient cells to differentiate into mature osteoblasts capable of forming a mineralized extracellular matrix. To this end, we treated Ad-GFP- or Ad-Cre-infected *Raptor* ^*f/f*^ calvarial cells with the osteogenic medium containing 10mM β-glycerol phosphate and 50μg/ml ascorbic acid to induce osteoblast differentiation and matrix mineralization. Von kossa staining detected mineralized bone nodules in control cells after 14 days of osteogenic inductions (Fig 2). In contrast, mineralized bone nodulesin Ad-Cre-infected cells were dramatically reduced (Fig 2). To assess bone matrix production, we examined the level of the amino terminal propeptide of type I procollagen(P1NP)in the culture medium, and found that the raptor-deficient cells produced significantly less P1NP (Fig 2). Alkaline phosphatase is known to regulate matrix mineralization. Therefore, in parallel experiments, we performed alkaline phosphatase staining. Surprisingly, *Raptor*-deficient cells exhibited slightly higher levels of alkaline phosphatase activity after7 days of the osteogenic induction (Fig 2). Therefore, deletion of raptor impaired matrix production and mineralization without reducing alkaline phosphatase activity.

### Raptor/mTORC1 pathway is dispensable for the early stages of osteoblast differentiation

**Fig 3:**
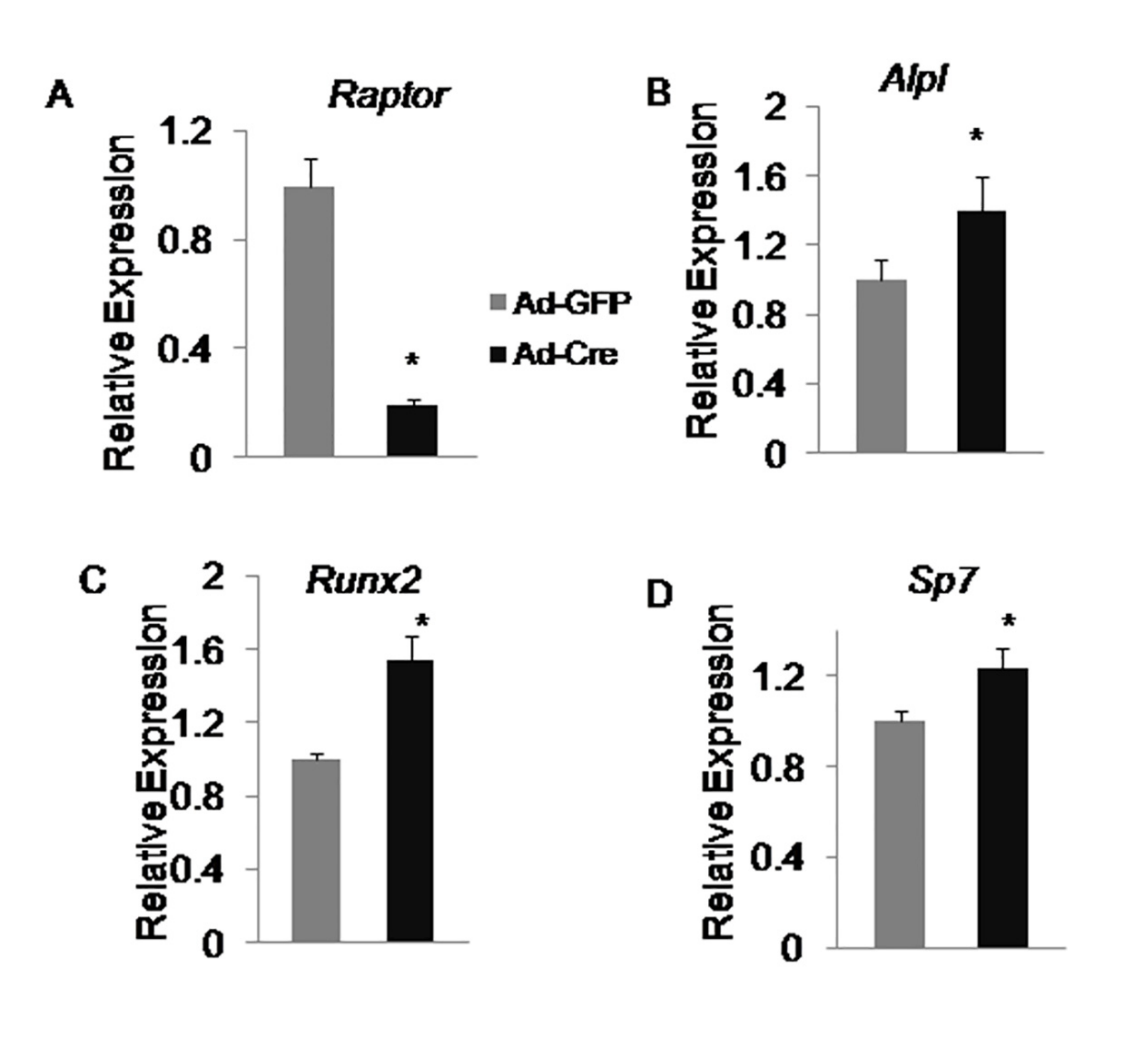
Raptor/mTORC1 pathway is dispensable for the early stages of osteoblast differentiation. qPCR analyses of early osteoblast markers in wildtype (Ad-GFP) versus *Raptor*-deficient (Ad-Cre) calvarial cells after 7 days (D7) of osteogenic differentiation. *: P<0.05, n = 3.

To determine whether the reduced bone nodules in *Raptor*-deficient cells was due to a defect in osteoblastogenesis, we first performed qPCR to analyze the expression of early markers for osteoblast differentiation (*Alpl*, *Runx2*, *and Sp7*). Consistent with the result from alkaline phosphatase staining, mRNA level of *Alpl*, a commonly used marker for early osteoblasts [22], was slightly but significantly increased in *Raptor*-deficient cells (Fig 3). Similarly, Runx2and Sp7, both transcriptional factors essential for osteoblast differentiation were higher in *Raptor*-deficient cells (Fig 3). In summary, these data indicate that *Raptor* is dispensable for the early steps of osteoblast differentiation.

### Raptor/mTORC1 pathway promotes the late stages of osteoblast differentiation

**Fig 4:**
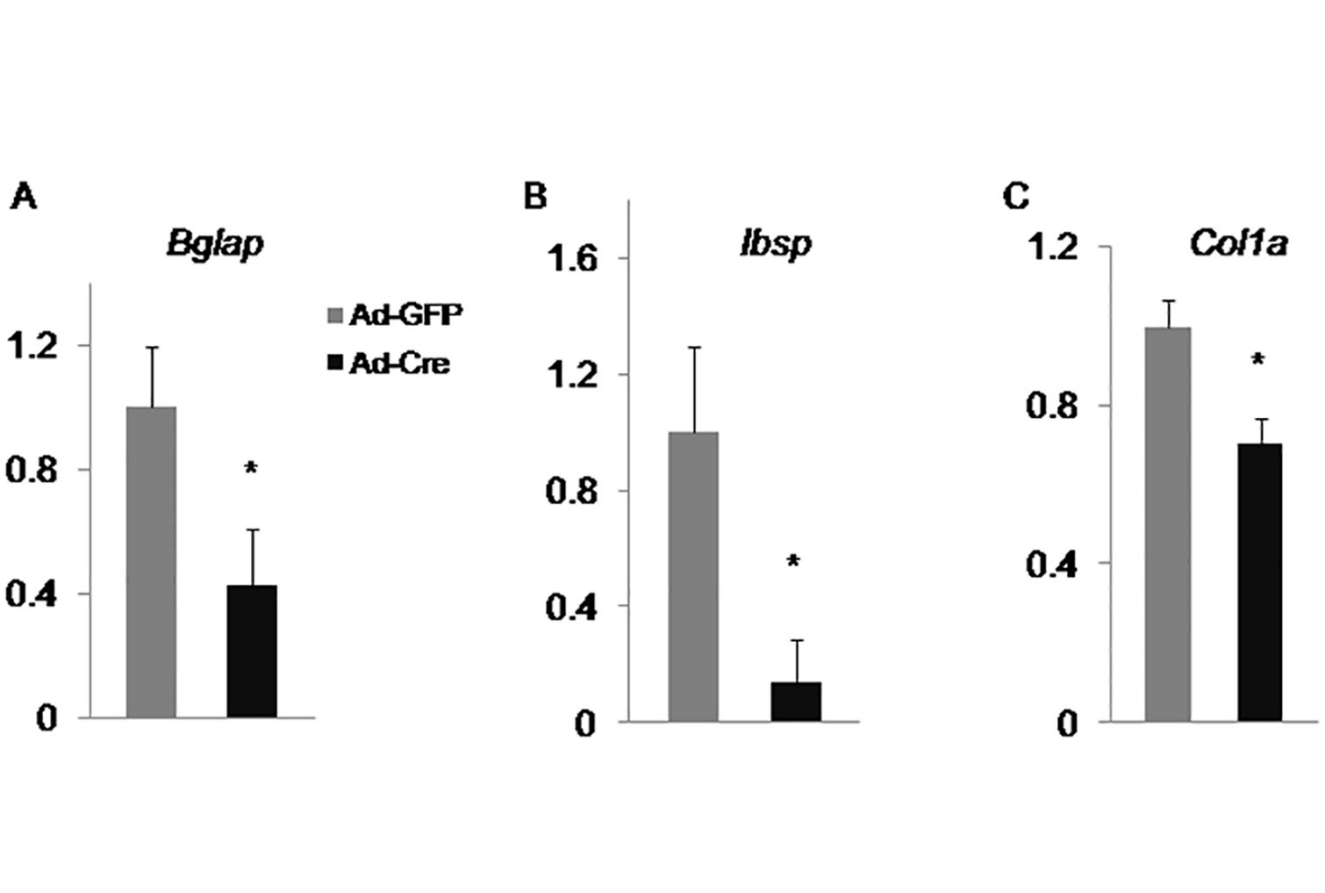
Raptor/mTORC1 pathway promotes the late stages of osteoblast differentiation. qPCR analyses of late osteoblast markers in wildtype (Ad-GFP) versus *Raptor*-deficient (Ad-Cre) calvarial cells after 7 days (D7) of osteogenic differentiation. *: P<0.05, n = 3.

Increased expression of early-stage markers for osteoblast differentiation in *Raptor*-deficient cells could be due to either enhanced osteoblastogenesis at all stages or arrest of differentiation at early stages. To distinguish these two possibilities, we examined expression of late stage markers for osteoblast differentiation, including *Bglap*, *Ibsp*, and *Col1a*. qPCR analysis revealed that both *Bglap* (encoding osteocalcin), a definitive marker for mature osteoblast, and *Ibsp* were markedly reduced whereas *Col1a* was mildly decreased in the mutant cells (Fig 4). Thus, raptor deletion caused a defect in the late stage of osteoblast differentiation.

### Reduced trabecular bone mass in *Osx-cre; Raptor* ^*f/f*^mice

**Fig 5:**
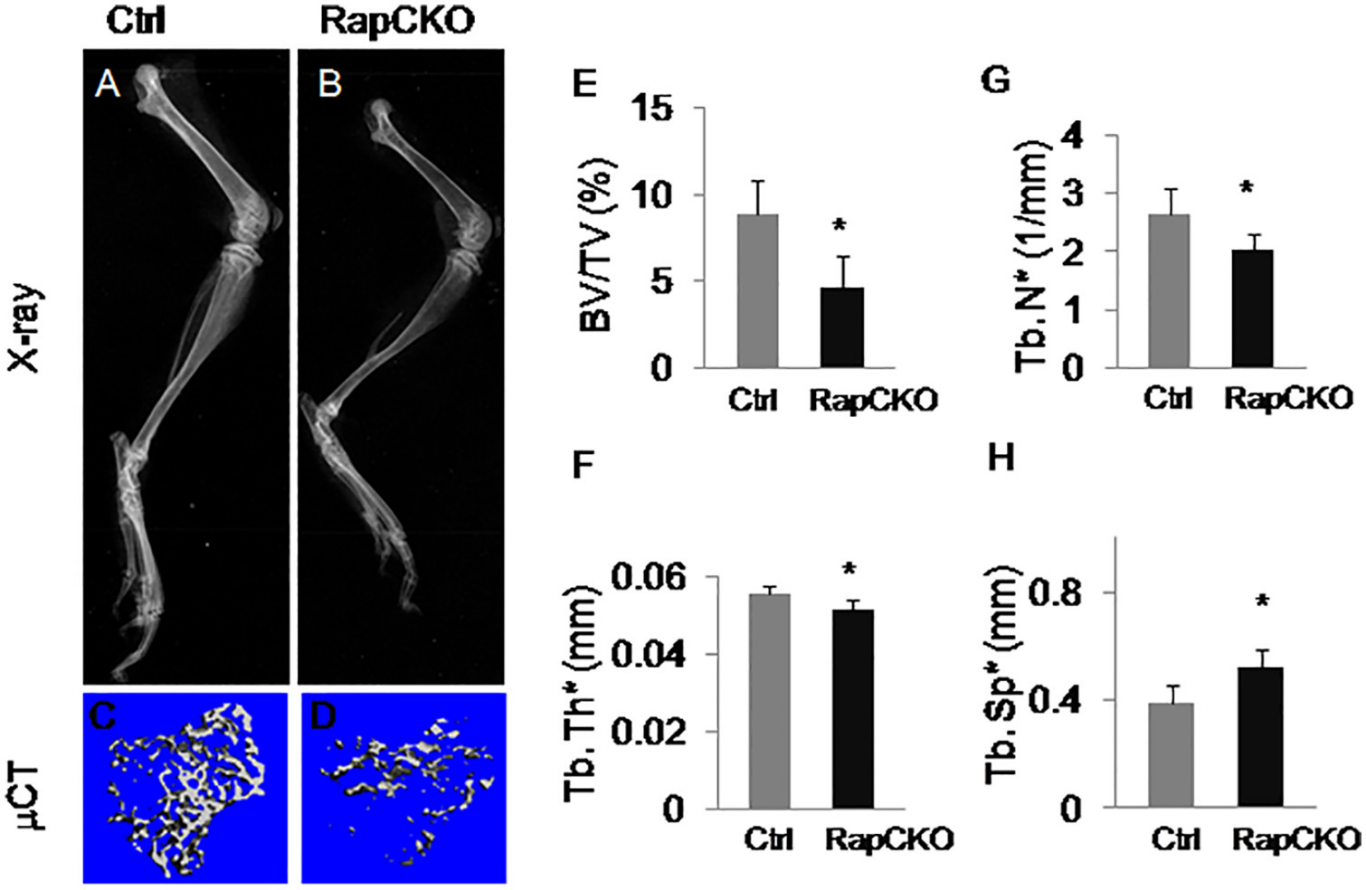
*Osx-cre; Raptor* ^*f/f*^mice exhibited reduced trabecular bone mass. (A-B) Representative X-ray images of hind limbs of 6 week-old *Osx-Cre* (Ctrl) (A) or *Osx-Cre; Raptor* ^*f/f*^(RapCKO) mice. (B). (C-D) μCT three-dimensional reconstruction of metaphyseal trabecular bone of the tibia from 6 week-old *Osx-Cre* (Ctrl) (C) or *Osx-Cre; Raptor* ^*f/f*^ (RapCKO) littermate mice(D). (E-H) μCT quantification of proximal metaphyseal trabecular bone parameters. BV: bone volume; TV: total volume; Tb. N*: trabecular number; Tb. Th*: trabecular thickness; Tb. Sp*: trabecular spacing. Quantification was performed on 100 of 16 μm slices immediately below the growth plate. Ctrl: n = 5; RapCKO: n = 4; *:P<0.05.

The data so far has demonstrated that mTORC1 plays a role in the formation of mature osteoblasts *in vitro*. To assess the role of mTORC1 in *vivo*, we generated *Osx-cre; Raptor* ^*f/f*^ conditional knockout mice to specifically disrupt mTORC1 in Osx-expressing cells. X-ray radiography indicated that mutant mice have slightly shorter bones compared to *Osx-Cre* mice (Control) (Fig 5). μCT analyses of the trabecular bone in the proximal tibias showed a significant reduction in bone mass (BV/TV) in 6 week-old *Osx-cre; Raptor* ^*f/f*^ mice when compared to control mice (Fig 5). The decrease in bone mass was due to both reduced trabecular number and trabecular thickness while accompanied by a significant increase in trabecular spacing (Fig 5). Our data suggested that Raptor/mTORC1 in Osx-expressing cells is important for promoting trabecular bone mass in mice.

**Fig 6:**
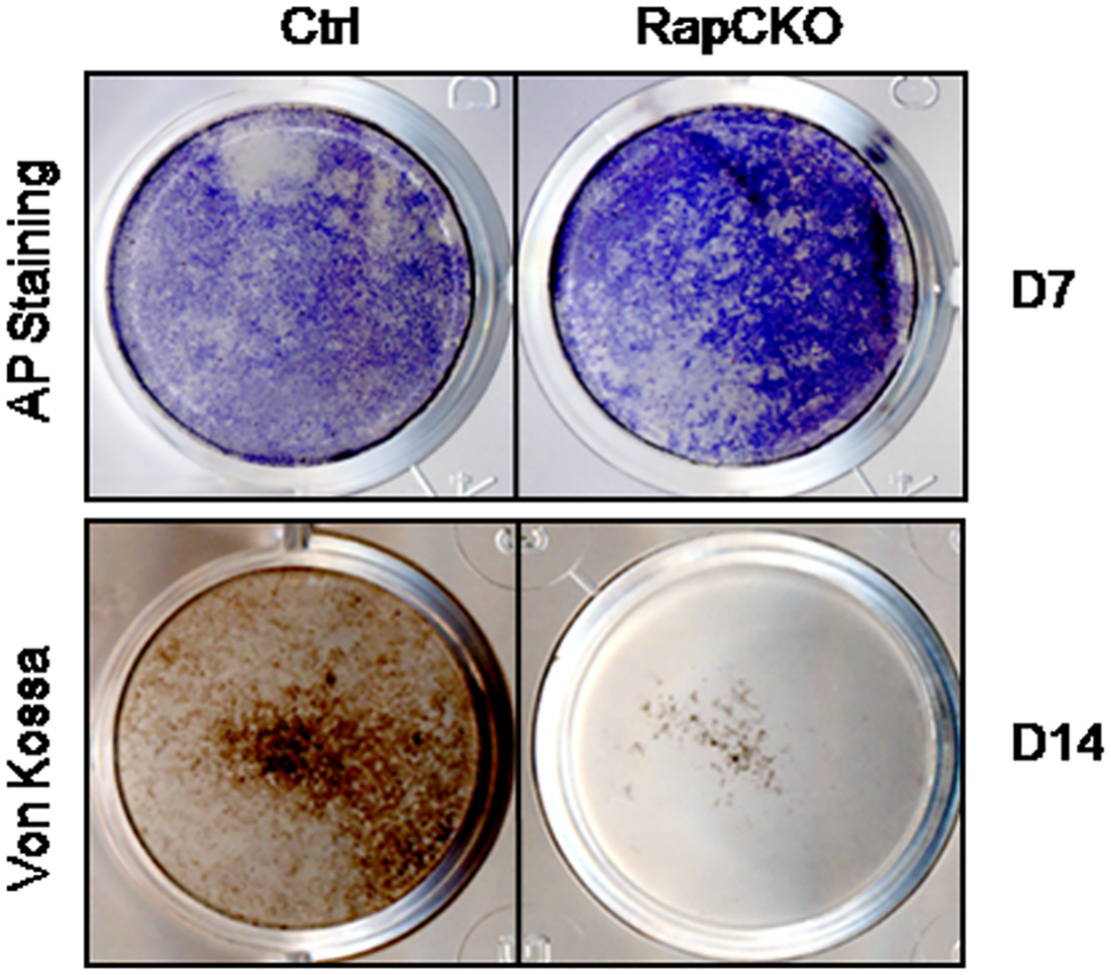
Bone marrow stromal cells isolated from *Osx-cre; Raptor* ^*f/f*^ mice exhibited reduced matrix synthesis and mineralization. (A-B) Alkaline phosphatase (AP) staining of bone marrow stromal cells isolated from *Osx-Cre* (Ctrl) (A) or *Osx-Cre; Raptor* ^*f/f*^(RapCKO) mice after 7 days (D7) of osteogenic differentiation. (C-D) Von Kossa staining of bone marrow stromal cells isolated from *Osx-Cre* (Ctrl) (A) or *Osx-Cre; Raptor* ^*f/f*^(RapCKO) mice after 14 days (D14) of osteogenic differentiation.

To further support a specific role of mTORC1 in osteoblast differentiation, we isolated bone marrow stromal cells from 6 week-old *Osx-cre; Raptor* ^*f/f*^ and control mice, and then treated them with osteogenic medium to induce osteoblast differentiation. Von Kossa staining detected abundant matrix mineralization in control cells after 14 days of osteognic induction, whereas BMSCs from *Osx-cre; Raptor* ^*f/f*^ mice largely failed to undergo mineralization, as evidenced by the lack of Von Kossa staining (Fig 6). In contrast, AP staining revealed that RapCKO BMSCs exhibited higher levels of alkaline phosphatase staining after 7 days of osteogenic induction (Fig 6). Collectively, these data suggested that reduced trabecular bone mass in *Osx-cre; Raptor* ^*f/f*^ mice is at least partially due to a defect in late stages of osteoblast differentiation.

## Discussion

Primary calvarial cells are commonly used as a cell culture system for studying osteoblast differentiation. Primary calvarial cells isolated from neonatal mice are mostly progenitor cells that are committed to the osteoblast lineage [1, 23]. By deleting *Raptor* in primary calvarial cells, our study revealed a critical role of mTORC1 in promoting osteoblast differentiation. In line with this, conditionally ablating *Raptor* in osterix-expressing cells in mice led to reduced trabecular bone mass. This suggested a role of mTORC1 in osteoblast differentiation in *vivo*. However, it should be mentioned that *Osx-cre* targets multiple cell types besides osteoblast lineage in postnatal mice [24]. Therefore, it still needs to be determined whether reduced trabecular bone mass in *Osx-cre; Raptor* ^*f/f*^ mice was caused solely by impaired osteoblast differentiation.

mTOR has been shown to be involved in regulating MPs lineage selection and osteoblast differentiation. However, mTOR participates in two different complexes, mTORC1 and mTORC2. Although a number of studies have demonstrated the pro-osteogenic role of mTORC2 in MPs [6, 25, 26, 27], the specific role played by mTORC1 in osteoblast differentiation is still controversial. By genetically deleting *Raptor* in MPs, a recent study showed that loss of *Raptor* promotes osteogenesis and inhibits adipogenesis [26]. Here, we showed that mTORC1 is important for late stages of osteoblast differentiation. The opposite results from these two studies may indicate that mTORC1 plays opposite roles in different stages of osteoblast differentiation. Future studies are warranted to test this possibility.

*Raptor*-deficient calvarial cells exhibit eda defect in osteoblast differentiation. Whether mTORC1 directly regulates osteoblast activity cannot be determined in the present study, asosteoblast activity is secondarily affected by impaired osteoblast differentiation. To address direct effects of mTORC1 on osteoblast activity, further studies should be performed on mature osteoblast.

Although mTORC1 is clearly involved in osteoblast differentiation, it is still unclear which downstream effector(s) mediate the function of mTORC1. Eukaryotic translation initiation factor 4E (eIF4E)-binding protein 1 (4E-BP1) and S6 kinase 1 (S6K1) are two major targets of mTORC1 pathway, both of which regulate protein synthesis [4]. It will be interesting to determine whether 4E-BP1 or S6K1 or both is the downstream mediator of mTORC1 in osteoblast differentiation.
